# Supplementary material for: Temporal relationship of suicide-related internet searches and suicide rates in Korea: A prewhitened cross-correlation analysis
Source: PLoS One. 2026 Feb 9;21(2):e0341656. doi: 10.1371/journal.pone.0341656 (PMC12885283; doi:10.1371/journal.pone.0341656)
Supplement: S1 Table — (DOCX) [file pone.0341656.s001.docx]

| **S1 Table.** Search terms examined in the study. |
| --- |
| Category: general |
| suicide (ja sal) |
| suicide urges (ja sal chung dong) |
| self-harm (ja hae) |
| suicide note (yu seo) |
| suicide death benefit (ja sal sa mang bo heom geum) |
| Category: method |
| suicide method (ja sal bang beop) |
| sleep pills (su myeon je) |
| charcoal briquette (beon gae tan) |
| jumping (tu shin) |
| pro-suicide website (ja sal sa i teu) |
| Category: reason |
| debt (bit) |
| unemployment (sil jik) |
| workplace stress (jik jang seu teu re seu) |
| divorce (yi hon) |
| bullying (wang tta) |
| Category: prevention |
| suicide crisis counseling (ja sal sang dam) |
| 1577-0199 |
| suicide prevention center (ja sal ye bang sen teo) |
| psychiatry (jeong sin geon gang ui hak gwa) |
| psychological counseling (sim ri sang dam) |
| Category: symptom |
| depression (woo ul) |
| anxiety (bul an) |
| insomnia (bul myeon) |
| loneliness (woe ro um) |
| fatigue (pi gon) |
| Terms in parentheses denote the Romanized Korean pronunciations of each search keyword as used for Naver search queries. |
