# Supplementary material for: Temporal relationship of suicide-related internet searches and suicide rates in Korea: A prewhitened cross-correlation analysis
Source: PLoS One. 2026 Feb 9;21(2):e0341656. doi: 10.1371/journal.pone.0341656 (PMC12885283; doi:10.1371/journal.pone.0341656)
Supplement: S2 Table — (DOCX) [file pone.0341656.s002.docx]

| **S2 Table.** Cross-correlation between weekly suicide-related search volumes (category: general) and suicide rates. | | | | | | | | | |
| --- | --- | --- | --- | --- | --- | --- | --- | --- | --- |
| Search term | lag 0 | lag 1 | lag 2 | lag 3 | lag 4 | lag 5 | lag 6 | lag 7 | lag 8 |
| Suicide | 2016 – 2019 | | | | | | | | |
|  | 0.186 | 0.046 | -0.034 | 0.036 | -0.009 | -0.020 | -0.006 | 0.152 | 0.066 |
|  | Fitted model : ARIMA(3,0,0) with non-zero mean; Ljung-Box test : Q* = 24.902, df = 39, P = 0.961 | | | | | | | | |
|  | 2020 – 2023 | | | | | | | | |
|  | 0.145 | 0.015 | -0.059 | 0.041 | 0.066 | 0.049 | -0.096 | 0.051 | 0.022 |
|  | Fitted model : ARIMA(1,1,1); Ljung-Box test : Q* = 29.805, df = 40, P = 0.881 | | | | | | | | |
| Suicide urges | 2016 – 2019 | | | | | | | | |
|  | 0.117 | -0.171 | 0.009 | -0.090 | 0.038 | -0.063 | 0.031 | 0.089 | -0.060 |
|  | Fitted model : ARIMA(1,1,2); Ljung-Box test : Q* = 30.005, df = 39, P = 0.849 | | | | | | | | |
|  | 2020 – 2023 | | | | | | | | |
|  | **0.261** | 0.024 | -0.053 | 0.037 | 0.144 | 0.025 | 0.015 | 0.071 | 0.007 |
|  | Fitted model : ARIMA(4,1,1); Ljung-Box test : Q* = 34.901, df = 37, P = 0.568 | | | | | | | | |
| Self-injury | 2016 – 2019 | | | | | | | | |
|  | 0.081 | -0.119 | 0.122 | 0.025 | -0.009 | 0.022 | 0.104 | 0.063 | 0.056 |
|  | Fitted model : ARIMA(5,1,0); Ljung-Box test : Q* = 27.788, df = 37, P = 0.864 | | | | | | | | |
|  | 2020 – 2023 | | | | | | | | |
|  | 0.090 | 0.054 | -0.074 | 0.034 | 0.056 | 0.035 | 0.031 | -0.047 | 0.049 |
|  | Fitted model : SARIMA(1,1,1)(1,1,0)[52]; Ljung-Box test : Q* = 29.25, df = 39, P = 0.872 | | | | | | | | |
| Suicide note | 2016 – 2019 | | | | | | | | |
|  | 0.072 | -0.092 | -0.107 | 0.078 | 0.001 | -0.047 | -0.050 | 0.153 | 0.063 |
|  | Fitted model : ARIMA(1,0,0) with non-zero mean; Ljung-Box test : Q* = 18.514, df = 41, P = 0.999 | | | | | | | | |
|  | 2020 – 2023 | | | | | | | | |
|  | 0.026 | -0.014 | -0.074 | -0.035 | -0.002 | 0.064 | -0.090 | -0.098 | -0.004 |
|  | Fitted model : ARIMA(0,0,0) with non-zero mean; Ljung-Box test : Q* = 28.781, df = 42, P = 0.940 | | | | | | | | |
| Suicide death benefit | 2016 – 2019 | | | | | | | | |
|  | 0.122 | -0.104 | -0.038 | 0.061 | -0.049 | -0.029 | 0.012 | 0.031 | 0.055 |
|  | Fitted model : ARIMA(3,0,0) with non-zero mean; Ljung-Box test : Q* = 28.089, df = 39, P = 0.903 | | | | | | | | |
|  | 2020 – 2023 | | | | | | | | |
|  | **0.303** | -0.086 | -0.043 | -0.023 | -0.007 | -0.014 | -0.038 | -0.017 | -0.025 |
|  | Fitted model : ARIMA(0,1,2); Ljung-Box test : Q* = 17.826, df = 40, P = 0.999 | | | | | | | | |
| Abbreviations: ARIMA, autoregressive integrated moving average; SARIMA, seasonal ARIMA  Cross-correlation analysis was performed between the residuals of the search volume and suicide rate time series after prewhitening. Lag is in weeks. Bold values denote significance at the Bonferroni-adjusted level (α=0.05/50; P<0.001). | | | | | | | | | |
