# Supplementary material for: Temporal relationship of suicide-related internet searches and suicide rates in Korea: A prewhitened cross-correlation analysis
Source: PLoS One. 2026 Feb 9;21(2):e0341656. doi: 10.1371/journal.pone.0341656 (PMC12885283; doi:10.1371/journal.pone.0341656)
Supplement: S3 Table — (DOCX) [file pone.0341656.s003.docx]

| **S3 Table.** Cross-correlation between weekly suicide-related search volumes (category: method) and suicide rates. | | | | | | | | | |
| --- | --- | --- | --- | --- | --- | --- | --- | --- | --- |
| Search term | lag 0 | lag 1 | lag 2 | lag 3 | lag 4 | lag 5 | lag 6 | lag 7 | lag 8 |
| Suicide method | 2016 – 2019 | | | | | | | | |
|  | 0.202 | -0.092 | -0.109 | 0.036 | -0.016 | -0.087 | -0.075 | 0.122 | 0.070 |
|  | Fitted model : ARIMA(2,0,1) with non-zero mean; Ljung-Box test : Q* = 21.697, df = 39, P = 0.989 | | | | | | | | |
|  | 2020 – 2023 | | | | | | | | |
|  | 0.139 | 0.064 | -0.101 | 0.050 | 0.064 | 0.067 | -0.086 | -0.013 | -0.011 |
|  | Fitted model : ARIMA(1,1,1) with non-zero mean; Ljung-Box test : Q* = 35.238, df = 40, P = 0.684 | | | | | | | | |
| Sleeping pills | 2016 – 2019 | | | | | | | | |
|  | 0.138 | -0.071 | -0.032 | 0.033 | -0.062 | 0.071 | -0.113 | 0.113 | -0.123 |
|  | Fitted model : ARIMA(1,1,1); Ljung-Box test : Q* = 32.424, df = 40, P = 0.797 | | | | | | | | |
|  | 2020 – 2023 | | | | | | | | |
|  | 0.133 | -0.001 | -0.068 | -0.135 | 0.033 | -0.064 | -0.012 | 0.020 | -0.115 |
|  | Fitted model : ARIMA(1,1,1); Ljung-Box test : Q* = 26.571, df = 40, P = 0.949 | | | | | | | | |
| Charcoal briquette | 2016 – 2019 | | | | | | | | |
|  | 0.116 | -0.134 | -0.148 | 0.075 | -0.078 | -0.076 | -0.026 | 0.041 | 0.084 |
|  | Fitted model : SARIMA(1,1,1)(0,0,1)[52]; Ljung-Box test : Q* = 13.335, df = 39, P = 1.000 | | | | | | | | |
|  | 2020 – 2023 | | | | | | | | |
|  | 0.199 | -0.153 | -0.093 | 0.041 | -0.139 | -0.051 | -0.014 | -0.146 | 0.001 |
|  | Fitted model : ARIMA(1,1,1); Ljung-Box test : Q* = 2.5225, df = 40, P = 1.000 | | | | | | | | |
| Jumping | 2016 – 2019 | | | | | | | | |
|  | 0.067 | 0.071 | -0.025 | 0.010 | 0.063 | 0.029 | 0.023 | -0.012 | 0.043 |
|  | Fitted model : ARIMA(0,0,2) with non-zero mean; Ljung-Box test : Q* = 37.063, df = 40, P = 0.603 | | | | | | | | |
|  | 2020 – 2023 | | | | | | | | |
|  | 0.069 | 0.073 | -0.084 | 0.139 | 0.069 | 0.034 | -0.067 | 0.037 | 0.037 |
|  | Fitted model : ARIMA(2,1,1); Ljung-Box test : Q* = 32.423, df = 39, P = 0.763 | | | | | | | | |
| Pro-suicide website | 2016 – 2019 | | | | | | | | |
|  | 0.083 | 0.129 | 0.093 | -0.100 | -0.095 | 0.015 | 0.024 | -0.020 | -0.140 |
|  | Fitted model : ARIMA(0,1,5); Ljung-Box test : Q* = 21.041, df = 37, P = 0.984 | | | | | | | | |
|  | 2020 – 2023 | | | | | | | | |
|  | 0.126 | -0.074 | 0.055 | -0.094 | 0.120 | -0.054 | -0.031 | 0.032 | 0.001 |
|  | Fitted model : ARIMA(1,1,1); Ljung-Box test : Q* = 51.757, df = 40, P = 0.101 | | | | | | | | |
| Abbreviations: ARIMA, autoregressive integrated moving average; SARIMA, seasonal ARIMA  Cross-correlation analysis was performed between the residuals of the search volume and suicide rate time series after prewhitening. Lag is in weeks. Bold values denote significance at the Bonferroni-adjusted level (α=0.05/50; P<0.001). | | | | | | | | | |
