# Supplementary material for: Temporal relationship of suicide-related internet searches and suicide rates in Korea: A prewhitened cross-correlation analysis
Source: PLoS One. 2026 Feb 9;21(2):e0341656. doi: 10.1371/journal.pone.0341656 (PMC12885283; doi:10.1371/journal.pone.0341656)
Supplement: S4 Table — (DOCX) [file pone.0341656.s004.docx]

| **S4 Table.** Cross-correlation between weekly suicide-related search volumes (category: reason) and suicide rates. | | | | | | | | | |
| --- | --- | --- | --- | --- | --- | --- | --- | --- | --- |
| Search term | lag 0 | lag 1 | lag 2 | lag 3 | lag 4 | lag 5 | lag 6 | lag 7 | lag 8 |
| Debt | 2016 – 2019 | | | | | | | | |
|  | 0.111 | -0.135 | 0.037 | 0.099 | -0.086 | 0.073 | -0.086 | -0.148 | -0.021 |
|  | Fitted model : SARIMA(1,1,1)(1,0,0)[52]; Ljung-Box test : Q* = 49.489, df = 39, p-value = 0.121 | | | | | | | | |
|  | 2020 – 2023 | | | | | | | | |
|  | 0.152 | -0.043 | 0.006 | -0.104 | 0.051 | -0.043 | -0.051 | 0.028 | 0.030 |
|  | Fitted model : ARIMA(1,1,1); Ljung-Box test : Q* = 40.015, df = 40, p-value = 0.470 | | | | | | | | |
| Unemployment | 2016 – 2019 | | | | | | | | |
|  | 0.106 | -0.024 | 0.083 | -0.044 | 0.050 | 0.107 | 0.049 | -0.017 | -0.031 |
|  | Fitted model : SARIMA(2,1,2)(1,0,0)[52]; Ljung-Box test : Q* = 47.295, df = 37, P = 0.120 | | | | | | | | |
|  | 2020 – 2023 | | | | | | | | |
|  | 0.041 | -0.110 | 0.067 | -0.066 | 0.086 | 0.032 | -0.068 | 0.018 | 0.132 |
|  | Fitted model : ARIMA(2,1,1); Ljung-Box test : Q* = 33.516, df = 39, P = 0.718 | | | | | | | | |
| Workplace stress | 2016 – 2019 | | | | | | | | |
|  | **0.232** | -0.033 | -0.101 | 0.006 | -0.090 | 0.107 | -0.097 | 0.017 | 0.017 |
|  | Fitted model : ARIMA(2,1,1); Ljung-Box test : Q* = 37.423, df = 39, P = 0.542 | | | | | | | | |
|  | 2020 – 2023 | | | | | | | | |
|  | **0.241** | -0.006 | 0.108 | -0.128 | 0.125 | -0.059 | -0.040 | -0.088 | 0.125 |
|  | Fitted model : ARIMA(0,1,2); Ljung-Box test : Q* = 47.238, df = 40, P = 0.201 | | | | | | | | |
| Divorce | 2016 – 2019 | | | | | | | | |
|  | -0.021 | -0.052 | 0.016 | 0.029 | 0.100 | -0.061 | 0.070 | -0.024 | -0.014 |
|  | Fitted model : ARIMA(0,1,2); Ljung-Box test : Q* = 37.937, df = 40, P = 0.564 | | | | | | | | |
|  | 2020 – 2023 | | | | | | | | |
|  | -0.090 | -0.026 | -0.025 | 0.048 | -0.001 | -0.063 | 0.057 | -0.123 | 0.120 |
|  | Fitted model : ARIMA(0,1,5); Ljung-Box test : Q* = 27.427, df = 37, P = 0.874 | | | | | | | | |
| Bullying | 2016 – 2019 | | | | | | | | |
|  | 0.032 | 0.085 | 0.030 | 0.019 | -0.037 | 0.162 | -0.005 | 0.010 | 0.028 |
|  | Fitted model : SARIMA(0,1,1)(1,1,0)[52]; Ljung-Box test : Q* = 34.628, df = 40, P = 0.710 | | | | | | | | |
|  | 2020 – 2023 | | | | | | | | |
|  | 0.093 | 0.024 | 0.114 | -0.179 | 0.129 | -0.061 | 0.034 | -0.036 | 0.059 |
|  | Fitted model : SARIMA(0,1,3)(1,0,0)[52]; Ljung-Box test : Q* = 28.753, df = 38, P = 0.861 | | | | | | | | |
| Abbreviations: ARIMA, autoregressive integrated moving average; SARIMA, seasonal ARIMA  Cross-correlation analysis was performed between the residuals of the search volume and suicide rate time series after prewhitening. Lag is in weeks. Bold values denote significance at the Bonferroni-adjusted level (α=0.05/50; P<0.001). | | | | | | | | | |
