# Supplementary material for: Temporal relationship of suicide-related internet searches and suicide rates in Korea: A prewhitened cross-correlation analysis
Source: PLoS One. 2026 Feb 9;21(2):e0341656. doi: 10.1371/journal.pone.0341656 (PMC12885283; doi:10.1371/journal.pone.0341656)
Supplement: S5 Table — (DOCX) [file pone.0341656.s005.docx]

| **S5 Table.** Cross-correlation between weekly suicide-related search volumes (category: prevention) and suicide rates. | | | | | | | | | |
| --- | --- | --- | --- | --- | --- | --- | --- | --- | --- |
| Search term | lag 0 | lag 1 | lag 2 | lag 3 | lag 4 | lag 5 | lag 6 | lag 7 | lag 8 |
| Suicide crisis counseling | 2016 – 2019 | | | | | | | | |
|  | 0.181 | -0.032 | -0.092 | 0.053 | -0.042 | -0.082 | -0.031 | 0.091 | 0.045 |
|  | Fitted model : ARIMA(0,1,1); Ljung-Box test : Q* = 31.511, df = 41, P = 0.857 | | | | | | | | |
|  | 2020 – 2023 | | | | | | | | |
|  | **0.266** | 0.064 | -0.045 | -0.010 | 0.078 | 0.120 | -0.037 | -0.008 | 0.035 |
|  | Fitted model : ARIMA(0,1,1); Ljung-Box test : Q* = 40.703, df = 41, P = 0.484 | | | | | | | | |
| 1577-0199 | 2016 – 2019 | | | | | | | | |
|  | 0.180 | -0.010 | -0.005 | 0.066 | 0.013 | -0.049 | 0.028 | 0.145 | 0.022 |
|  | Fitted model : ARIMA(2,1,1); Ljung-Box test : Q* = 32.343, df = 39, P = 0.766 | | | | | | | | |
|  | 2020 – 2023 | | | | | | | | |
|  | **0.238** | 0.126 | -0.027 | 0.036 | 0.011 | -0.005 | -0.025 | 0.077 | -0.011 |
|  | Fitted model : ARIMA(1,1,2); Ljung-Box test : Q* = 36.079, df = 39, P = 0.604 | | | | | | | | |
| Suicide prevention center | 2016 – 2019 | | | | | | | | |
|  | 0.189 | 0.068 | 0.019 | 0.032 | 0.021 | 0.147 | -0.144 | 0.008 | -0.016 |
|  | Fitted model : ARIMA(1,1,3); Ljung-Box test : Q* = 40.197, df = 38, P = 0.373 | | | | | | | | |
|  | 2020 – 2023 | | | | | | | | |
|  | 0.201 | 0.037 | 0.070 | -0.140 | 0.078 | 0.057 | -0.158 | 0.064 | -0.005 |
|  | Fitted model : ARIMA(1,1,1); Ljung-Box test : Q* = 46.761, df = 40, P = 0.215 | | | | | | | | |
| Psychiatry | 2016 – 2019 | | | | | | | | |
|  | **0.250** | -0.030 | -0.036 | -0.052 | -0.032 | 0.099 | -0.039 | -0.048 | 0.100 |
|  | Fitted model : ARIMA(1,1,1); Ljung-Box test : Q* = 37.646, df = 40, P = 0.577 | | | | | | | | |
|  | 2020 – 2023 | | | | | | | | |
|  | **0.266** | -0.023 | 0.045 | -0.037 | 0.175 | -0.003 | -0.019 | -0.040 | -0.048 |
|  | Fitted model : ARIMA(0,1,1); Ljung-Box test : Q* = 38.486, df = 41, P = 0.583 | | | | | | | | |
| Psychological counseling | 2016 – 2019 | | | | | | | | |
|  | 0.179 | -0.005 | 0.045 | 0.071 | -0.161 | 0.029 | -0.061 | -0.038 | 0.035 |
|  | Fitted model : SARIMA(3,1,0)(1,0,0)[52]; Ljung-Box test : Q* = 48.833, df = 38, P = 0.112 | | | | | | | | |
|  | 2020 – 2023 | | | | | | | | |
|  | **0.255** | -0.054 | 0.164 | -0.199 | 0.181 | -0.020 | -0.102 | -0.009 | 0.016 |
|  | Fitted model : ARIMA(0,1,2); Ljung-Box test : Q* = 43.402, df = 40, P = 0.329 | | | | | | | | |
| Abbreviations: ARIMA, autoregressive integrated moving average; SARIMA, seasonal ARIMA  Cross-correlation analysis was performed between the residuals of the search volume and suicide rate time series after prewhitening. Lag is in weeks. Bold values denote significance at the Bonferroni-adjusted level (α=0.05/50; P<0.001). | | | | | | | | | |
