# Supplementary material for: Temporal relationship of suicide-related internet searches and suicide rates in Korea: A prewhitened cross-correlation analysis
Source: PLoS One. 2026 Feb 9;21(2):e0341656. doi: 10.1371/journal.pone.0341656 (PMC12885283; doi:10.1371/journal.pone.0341656)
Supplement: S6 Table — (DOCX) [file pone.0341656.s006.docx]

| **S6 Table.** Cross-correlation between weekly suicide-related search volumes (category: symptom) and suicide rates. | | | | | | | | | |
| --- | --- | --- | --- | --- | --- | --- | --- | --- | --- |
| Search term | lag 0 | lag 1 | lag 2 | lag 3 | lag 4 | lag 5 | lag 6 | lag 7 | lag 8 |
| Depression | 2016 – 2019 | | | | | | | | |
|  | **0.303** | -0.080 | -0.055 | 0.088 | 0.005 | -0.067 | -0.042 | 0.110 | 0.051 |
|  | Fitted model : ARIMA(0,1,1); Ljung-Box test : Q* = 24.112, df = 41, P = 0.984 | | | | | | | | |
|  | 2020 – 2023 | | | | | | | | |
|  | 0.147 | 0.027 | 0.060 | 0.007 | 0.011 | 0.006 | -0.034 | 0.065 | 0.116 |
|  | Fitted model : ARIMA(0,1,1); Ljung-Box test : Q* = 17.91, df = 41, P = 0.999 | | | | | | | | |
| Anxiety | 2016 – 2019 | | | | | | | | |
|  | 0.189 | 0.002 | 0.170 | -0.073 | 0.079 | 0.143 | -0.051 | 0.067 | -0.054 |
|  | Fitted model : SARIMA(2,0,2)(1,1,0)[52] with drift; Ljung-Box test : Q* = 35.787, df = 37, P = 0.526 | | | | | | | | |
|  | 2020 – 2023 | | | | | | | | |
|  | 0.226 | -0.088 | 0.125 | -0.150 | 0.076 | -0.020 | -0.056 | 0.078 | 0.021 |
|  | Fitted model : SARIMA(0,1,2)(1,1,0)[52]; Ljung-Box test : Q* = 49.055, df = 39, P = 0.130 | | | | | | | | |
| Insomnia | 2016 – 2019 | | | | | | | | |
|  | 0.090 | 0.005 | -0.025 | 0.045 | 0.059 | 0.017 | 0.037 | -0.065 | 0.081 |
|  | Fitted model : ARIMA(2,1,1); Ljung-Box test : Q* = 21.505, df = 39, P = 0.990 | | | | | | | | |
|  | 2020 – 2023 | | | | | | | | |
|  | 0.050 | -0.116 | -0.094 | -0.065 | -0.066 | 0.032 | -0.067 | -0.043 | 0.105 |
|  | Fitted model : ARIMA(0,1,3); Ljung-Box test : Q* = 22.116, df = 39, P = 0.987 | | | | | | | | |
| Loneliness | 2016 – 2019 | | | | | | | | |
|  | 0.040 | -0.063 | 0.091 | -0.030 | 0.084 | -0.043 | -0.031 | 0.103 | 0.002 |
|  | Fitted model : ARIMA(3,1,2); Ljung-Box test : Q* = 48.127, df = 37, P = 0.104 | | | | | | | | |
|  | 2020 – 2023 | | | | | | | | |
|  | 0.128 | 0.097 | 0.021 | -0.017 | -0.009 | -0.016 | 0.130 | -0.130 | 0.059 |
|  | Fitted model : ARIMA(1,0,1) with non-zero mean; Ljung-Box test : Q* = 32.304, df = 40, P = 0.802 | | | | | | | | |
| Fatigue | 2016 – 2019 | | | | | | | | |
|  | 0.191 | 0.046 | 0.031 | 0.066 | 0.053 | 0.099 | -0.063 | 0.003 | -0.028 |
|  | Fitted model : SARIMA(1,1,1)(1,1,0)[52]; Ljung-Box test : Q* = 32.934, df = 39, P = 0.742 | | | | | | | | |
|  | 2020 – 2023 | | | | | | | | |
|  | **0.391** | -0.027 | 0.188 | -0.076 | 0.063 | 0.052 | -0.047 | 0.025 | 0.032 |
|  | Fitted model : SARIMA(0,1,2)(1,1,0)[52]; Ljung-Box test : Q* = 35.027, df = 39, P = 0.652 | | | | | | | | |
| Abbreviations: ARIMA, autoregressive integrated moving average; SARIMA, seasonal ARIMA  Cross-correlation analysis was performed between the residuals of the search volume and suicide rate time series after prewhitening. Lag is in weeks. Bold values denote significance at the Bonferroni-adjusted level (α=0.05/50; P<0.001). | | | | | | | | | |
